# Polymorphism analysis of miR182 and CDKN2B genes in Greek patients with primary open angle glaucoma

**DOI:** 10.1371/journal.pone.0233692

**Published:** 2020-06-03

**Authors:** Marilita M. Moschos, Maria Dettoraki, Aggela Karekla, Ioannis Lamprinakis, Christos Damaskos, Nikolaos Gouliopoulos, Marios Tibilis, Maria Gazouli

**Affiliations:** 1 1st Department of Ophthalmology, "G. Gennimatas" General Hospital, Medical School, National and Kapodistrian University of Athens, Athens, Greece; 2 Department of Ophthalmology, “Evangelismos” General Hospital, Athens, Greece; 3 Second Department of Propedeutic Surgery, “Laiko” General Hospital, Medical School, National and Kapodistrian University of Athens, Athens, Greece; 4 Department of Basic Medical Sciences, Laboratory of Biology, Medical School, National and Kapodistrian University of Athens, Athens, Greece; University of Florida, UNITED STATES

## Abstract

Glaucoma is a progressive optic neuropathy resulting from retinal ganglion cells death; it represents one of the leading causes of irreversible blindness worldwide. Although, primary open angle glaucoma (POAG) is the most common type of the disease, the pathogenesis of POAG and the genetic factors contributing to disease development remain poorly understood. The aim of this study was to investigate whether the polymorphisms rs76481776 in miR182 gene and rs3217992 in cyclin-dependent kinase inhibitor-2B (CDKN2B) gene are risk factors for POAG in a series of patients of Greek origin. A case-control study was conducted including 120 patients with POAG and 113 unaffected healthy controls of Greek origin, surveyed for polymorphisms with potential correlation to POAG. DNA from each individual was tested for the miR182 rs76481776 and CDKN2B rs3217992 polymorphisms. Regarding the miR182 rs76481776 polymorphism, the T allele occurred with significantly higher frequency in POAG patients compared to controls (OR: 2.62, 95% CI: 1.56–4.39; p = 0.0002). The CDKN2B rs3217992 A allele frequency was found significantly increased in POAG patients compared to healthy individuals (OR: 1.72, 95% CI: 1.18–2.49; p = 0.005). Therefore, both rs76481776 polymorphism in miR182 gene and rs3217992 polymorphism in CDKN2B gene seem to be associated with the development of POAG in a Greek population. The carriers of the T allele of rs76481776 in miR182 and the carriers of the A allele of rs3217992 in CDKN2B have an increased risk of developing POAG.

## Introduction

Glaucoma represents one of the leading causes of irreversible blindness worldwide. [1] Epidemiological studies suggest that the prevalence of the disease is estimated to have reached 76 million in 2020 and to increase to 111.8 million by 2040 globally due to the population aging. [1] Glaucoma is characterized by progressive optic neuropathy resulting from retinal ganglion cells death. [2] Intraocular pressure (IOP) elevation, myopia, vascular factors, aging and positive family history are considered major risk factors for glaucoma. [3] Although, primary open angle glaucoma (POAG) represents the most common type of the disease, the pathogenesis of POAG and factors contributing to disease progression remain poorly understood.

Genetic studies have established three POAG susceptibility genes: myocilin (MYOC), optineurin (OPTN) and WD repeat domain 36 (WDR36). [4–6] However, these genes are responsible for less than 10% of sporadic POAG cases. Genome-wide association studies (GWAS) have revealed several single nucleotide polymorphisms (SNPs) at different loci associated with glaucoma, including caveolin 1 (CAV1), caveolin 2 (CAV2), cyclin-dependent kinase inhibitor 2B-antisense noncoding RNA (CDKN2B-AS1), neurotrophin-4 (NTF4), transmembrane and coiled-coil domains 1 (TMCO1) and more recently, thioredoxin reductase 2 (TXNRD2), ataxin 2 (ATXN2), forkhead box C1 (FOXC1), SIX6, GLI-similar 3 (GLIS3), fibronectin type III domain containing 3B (FNDC3B), LIM homeobox transcription factor 1B (LMX1B), phospholysine phosphohistidine inorganic pyrophosphate phosphatase (LHPP), myeloid ecotropic insertion site 2 (MEIS2) and lysyl oxidase like 1 (LOXL1). [7–15]

MicroRNAs (miRNAs) are small, non-coding RNAs consisting of about 22 nucleotides that regulate gene expression at the post-transcriptional level, affecting a variety of biological processes. [16] Growing evidence suggests that miRNAs play an important role in the pathogenesis of POAG. Specifically, miR-93 induces apoptosis in trabecular meshwork cells, [17] miR-187 inhibits the oxidative stress-induced retinal ganglion cells apoptosis and promotes cell proliferation [18, 19] and miR-183 targets integrin-β1 and affects trabecular meshwork physiology. [20] miR-29b, regulated by TGF-β2, modulates the expression of extracellular matrix genes, which function in the aqueous outflow pathway. [21]

Previous studies have shown that sequence alterations in MIR genes encoding miRNAs have profound effects on miRNA biogenesis and function and contribute to the development risk of various diseases. [22–25] For example, hsa-miR-568 polymorphism has been identified in sporadic keratoconus and a mutation altering the miR-184 seed region has been reported to cause keratoconus with congenital cataract. [26, 27] Genetic variants of miR-146a are associated with pediatric uveitis and mutations in miR-182 predispose to Behcet’s disease and Vogt-Koyanagi-Harada syndrome. [28, 29] Variants in several MIR genes have been also associated with POAG. Recently, variants in the miR-612 precursor and in the miR-4707 seed region were found to be significantly associated with vertical cup-to-disc ratio and cup area. [30] In addition, polymorphisms in miR-3196 and miR-182 have been associated with POAG. [31, 32]

The CDKN2B gene encodes a cyclin-dependent kinase inhibitor, which plays a pivotal role in the maintenance of cell cycle progression. [33] Animal studies have shown that elevated IOP is associated with overexpression of CDKN2B, leading to disruption in cell cycle and abnormal cell proliferation. [34] Additionally, chromosome 9p21, where the CDKN2B gene is located, has been identified as an important susceptibility locus for glaucoma with various SNPs having positive associations with POAG in different ethnic populations. [9, 35, 36] Both rs3217992 and rs1063192 polymorphisms in CDKN2B gene are miRNA-binding-site variants, with the association of rs1063192 with POAG being widely studied in various ethnic groups. [30, 35, 37] However, the possible association between CDKN2B rs3217992 polymorphism and glaucoma risk has not been studied in any population.

The aim of our study was to investigate the possible association between miR182 rs76481776 and CDKN2B gene rs3217992 polymorphisms and POAG susceptibility in a well-defined Greek cohort. To our knowledge, this is the first study in the literature to conduct such genotyping in Greek patients with POAG.

## Materials and methods

### Study population

Greek patients with POAG (n = 120) and 113 unaffected healthy controls with no history of any ophthalmologic or other systematic disease were recruited in the study at Glaucoma Unit of the First Department of Ophthalmology, National and Kapodistrian University of Athens, Athens, Greece. The diagnosis of POAG was clinically confirmed at least 10 years before recruitment in the study. All participants signed an informed consent and had a blood sample taken for genetic analysis. The study was approved by the Ethics committee of “Evangelismos” General Hospital of Athens (Director Mr Lamprinakis Ioannis, approval number 19877).

All participants were selected after ophthalmological evaluation and clinical data collection. Ophthalmological evaluation included slit lamp examination with gonioscopy, fundus examination for cup/disc ratio evaluation of the optic disc and IOP measurement with Goldmann applanation tonometer (Haag Streit AG, Bern, Switzerland). Automated perimetry with a Humphrey Automated Field Analyzer (Humphrey Inc., San Leandro, CA, USA) using 30–2 SITA protocol was performed at both cases and controls.

Inclusion criteria for the POAG patient group were the following: glaucomatous optic neuropathy in at least one eye, defined as a cup/disc ratio ≥0.6 on fundoscopy and repeatable glaucomatous visual field defect on automated perimetry, a Schaffer III-IV angle on gonioscopy and initial IOP ≥21mmHg (high tension POAG). The glaucomatous visual field defects included a Bjerrum, altitudinal or nasal step scotoma. The control group had no family history of glaucoma, normal IOP values (<21mmHg) and no glaucomatous defects were detected at the ophthalmological examination and automated perimetry.

### Genotyping

Genomic DNA was isolated from peripheral blood leukocytes using a commercial kit (Nucleospin Blood, Macherey-Nagel, Germany) according to the manufacturer’s instructions. The rs76481776 polymorphism was analyzed using PCR followed by restriction analysis, as previously described. [28] Regarding the rs3731249, the genotyping analyses were also performed by using PCR followed by restriction analysis, as previously described. [38]

#### Statistical analysis

Genotype frequencies were analyzed with the χ^2^ test with Yate’s correction using S-Plus (v.6.2 Insightful, Seattle, WA, United States) software. Odds ratios (OR) and 95% confidence intervals (95% CI) were calculated with GraphPad (v.300, GraphPad Software, San Diego, CA, United States). Hardy-Weinberg Equilibrium (HWE) deviation was tested by Pearson’s χ^2^ test. All p values are two-sided. A p value <0.05 was considered significant.

## Results

The demographic and clinical characteristics of the study population are shown in Table 1. Among POAG patients, 48 (40%) were male and 72 (60%) were female, with a mean age of 73, 4±11.5 years. Among controls, 58 (51%) were male and 55 (49%) were female, with a mean age of 59, 3±18.6 years. Controls were statistically significant younger than POAG patients (p<0.005). POAG patients had a mean IOP of 17.2±4mmHg, all were receiving anti-glaucoma medications and had a mean therapy duration of 7, 2±6.7 years.

**Table 1:** Demographics and clinical characteristics of the study population.

Variables	Controls (n = 113)	POAG (n = 120)	P value
Sex (male/female)	58/55	48/72	0.08
Age (mean±SD), years	59.3±18.6	73.4±11.5	<0.005
IOP (mean±SD), mmHg	14.3±2	17.2±4	<0.001
Duration of therapy (mean±SD), years	-	7.2±6.7	-

POAG, primary open-angle glaucoma; IOP, intraocular pressure

The genotype and allele frequency distribution of the rs76481776 miR182 gene and rs3217992 CDKN2B gene across controls and POAG patients is shown in Table 2. The miR182 rs76481776 CT genotype frequency was significantly higher in patients with POAG than in controls (30.83% and 15.93% respectively, OR:2.59, 95% CI:1.36–4.92; p = 0.005), as well as the TT genotype was significantly overrepresented in POAG patients compared to controls (8.33% and 2.65% respectively, OR: 4.2, 95% CI: 1.11–15.83; p = 0.04). The T allele of rs76481776 occurred with significantly higher frequency in POAG patients (OR: 2.62, 95% CI: 1.56–4.39; p = 0.0002).

**Table 2:** Genotype and allele frequency distribution of the rs76481776 miR182 gene and rs3217992 CDKN2B gene across controls and POAG patients.

Polymorphism	Genotype	Controls (n = 113)	POAG (n = 120)	OR (95% CI)	P value
*MIR182 rs76481776*	CC	92 (81.42%)	73 (60.83%)	1.00	
	CT	18 (15.93%)	37 (30.83%)	2.59 (1.36–4.92)	**0.005**
	TT	3 (2.65%)	10 (8.33%)	4.2 (1.11–15.83)	**0.04**
	C allele	202 (89.38%)	183 (73.25%)	1.00	
	T allele	24 (10.62%)	57 (23.75%)	2.62 (1.56–4.39)	**0.0002**
*CDKN2B rs3217992*	GG	49 (43.36%)	28 (23.33%)	1.00	
	GA	50 (44.25%)	70 (58.33%)	2.45 (1.36–4.42)	**0.003**
	AA	14 (12.39%)	22 (18.33%)	2.75 (1.22–6.21)	**0.016**
	G allele	148 (65.49%)	126 (52.5%)	1.00	**0.005**
	A allele	78 (34.51%)	114 (47.5%)	1.72 (1.18–2.49)	

POAG, primary open-angle glaucoma; OR, odds ratio; 95% CI, 95% confidence interval, p value≤0.05

Regarding the CDKN2B rs3217992 polymorphism, the GA genotype was significantly increased in POAG patients compared to controls (58.33% and 44.24% respectively, OR: 2.45, 95% CI: 1.36–4.42; p = 0.003). The AA genotype frequency was also significantly higher in patients with POAG than in controls (18.33% and 12.39%, respectively, OR: 2.75, 95% CI: 1.22–6.21; p = 0.016). The minor A allele frequency was higher in POAG patients (OR: 1.72, 95% CI: 1.18–2.49; p = 0.005).

## Discussion

POAG is a progressive neurodegenerative disorder induced by a combination of multiple genetic and environmental factors. [39] This study aimed to investigate the possible association of miR182 gene rs76481776 and CDKN2B gene rs3217992 polymorphisms with POAG susceptibility. To our knowledge, the genetic contribution of variants at the miR182 and CDKN2B loci among Greek POAG patients is not known. Our results demonstrated a significant association of the T allele of the rs76481776 miR182 gene with POAG, suggesting that the carriers of the T allele have an increased risk of developing POAG. Moreover, we showed an increased frequency of the CDKN2B rs3217992 A allele in POAG patients compared to controls, indicating that individuals carrying the A allele of the rs3217992 polymorphism are associated with the development of POAG in our population.

Concerning the miR182 gene, our results are in agreement with a previous study, [31] which identified a significant association of rs76481776 in miR182 gene with POAG in subjects with European ancestry from the United States. The study also reported an elevated expression of miR182 in ciliary body, trabecular meshwork and aqueous humor samples from patients with high-tension glaucoma compared to those from unaffected controls. The authors suggested a potential contribution of miR182 in POAG pathogenesis, probably through regulation of aqueous humor dynamics and IOP. Another research from Li and colleagues revealed a 7- to 9-fold upregulation of miR182 expression in primary cultures of human trabecular meshwork cells during stress-induced premature senescence, which may contribute to the phenotypic alterations of senescent cells. [40] It is hypothesized that miR182 overexpression in trabecular meshwork cells may lead to potential cellular dysfunction, such as cell contractility and phagocytosis ability [31] and may underlie, as a consequence, the increase in glaucoma risk. Interestingly, the minor T allele of the rs76481776, which is overrepresented in our POAG patients, has been previously reported to increase the expression of mature miR-182 in vitro. [25] However, there is still little information regarding the involvement of miR182 and its polymorphisms in the pathophysiology of glaucoma and more research is needed for a better understanding of their role in glaucoma development.

CDKN2B is a tumor suppressor gene involved in cell signaling pathways and polymorphisms in this gene have been associated with various diseases, including multiple cancers, [41] gestational diabetes mellitus, [42] familial combined hyperlipidemia [43] and glaucoma. [44–49] The gene is located at the chromosome 9q21, which is considered a strong candidate for POAG risk. [36] Several studies have investigated the association of SNP rs1063192, a common variant near CDKN2B, with POAG in different ethnic groups but with conflicting findings. [9, 35, 36] The small sample sizes, the clinical heterogeneity and the different ethnic populations seem to be the main factors contributing to the inconclusive findings of the studies. [37] A recent meta-analysis of Hu and He [37] found that rs1063192 polymorphism decreases the risk of glaucoma among Caucasians, Asians and Africans. However, the SNP of CDKN2B rs3217992 in patients with glaucoma has not been genotyped until now. Our study demonstrated an association between the CDKN2B rs3217996 polymorphism and the development of POAG in a Greek population. Both rs3217992 and rs1063192 polymorphisms are considered miRNA-binding-site variants that has been shown to affect the miRNA-mediated regulation of the CDKN2B gene in vitro. [43, 50] It has been shown that rs3217992 and rs1063192 affect miR-138-3p- and miR-323b-5p-mediated regulation of CDKN2B respectively and an allelic-specific regulation in these miRNA-binding sites is considered a potential mechanism, at least in part, to explain the association between the CDKN2B polymorphisms and POAG. [30, 50]

Limitations of our study include the small sample size, the small number of SNPs and the difference concerning the age between the two groups of the study.

In conclusion, our study demonstrated that rs76481776 in miR182 gene and rs3217992 in CDKN2B gene are associated with the development of POAG in a Greek population. Specifically, the carriers of the T allele of rs76481776 in miR182 and the carriers of the A allele of rs3217992 in CDKN2B have an increased risk of developing POAG in our population. Taking into consideration the complexity of the disease pathogenesis and the variety of the genetic and environmental factors involved, future studies of populations with different ethnicities are needed to investigate the association of these polymorphisms with POAG risk.

## Supporting information

S1 Data (XLSX)

## References

[1] Quigley HA and Broman AT. The number of people with glaucoma worldwide in 2010 and 2020. Br J Ophthalmol. 2006;90: 262–267. 10.1136/bjo.2005.081224 16488940 PMC1856963

[2] Weinreb RN and Khaw PT. Primary open-angle glaucoma. Lancet. 2004;363: 1711–1720. 10.1016/S0140-6736(04)16257-0 15158634

[3] Worley A and Grimmer-Somers K. Risk factors for glaucoma: what do they really mean? Aust J Prim Health. 2011;17: 233–239. 10.1071/PY10042 21896259

[4] Alward WL, Kwon YH, Kawase K, Craig JE, Hayreh SS, Johnson AT, et al Evaluation of optineurin sequence variations in 1,048 patients with open-angle glaucoma. Am J Ophthalmol. 2003;136: 904–910. 10.1016/s0002-9394(03)00577-4 14597044

[5] Fingert JH, Heon E, Liebmann JM, Yamamoto T, Craig JE, Rait J, et al Analysis of myocilin mutations in 1703 glaucoma patients from five different populations. Hum Mol Genet. 1999;8: 899–905. 10.1093/hmg/8.5.899 10196380

[6] Hewitt AW, Dimasi DP, Mackey DA, Craig JE. A Glaucoma Case-control Study of the WDR36 Gene D658G sequence variant. Am J Ophthalmol. 2006;142: 324–325. 10.1016/j.ajo.2006.02.041 16876519

[7] Chen Y, Hughes G, Chen X, Qian S, Cao W, Wang L, et al Genetic Variants Associated With Different Risks for High Tension Glaucoma and Normal Tension Glaucoma in a Chinese Population. Invest Ophthalmol Vis Sci. 2015;56: 2595–2600. 10.1167/iovs.14-16269 25711633

[8] Loomis SJ, Kang JH, Weinreb RN, Yaspan BL, Cooke Bailey JN, Gaasterland D, et al Association of CAV1/CAV2 genomic variants with primary open-angle glaucoma overall and by gender and pattern of visual field loss. Ophthalmology. 2014;121: 508–516. 10.1016/j.ophtha.2013.09.012 24572674 PMC3937766

[9] Pasquale LR, Loomis SJ, Kang JH, Yaspan BL, Abdrabou W, Budenz DL, et al CDKN2B-AS1 genotype-glaucoma feature correlations in primary open-angle glaucoma patients from the United States. Am J Ophthalmol. 2013;155: 342–53 e5. 10.1016/j.ajo.2012.07.023 23111177 PMC3544983

[10] Vithana EN, Nongpiur ME, Venkataraman D, Chan SH, Mavinahalli J, Aung T. Identification of a novel mutation in the NTF4 gene that causes primary open-angle glaucoma in a Chinese population. Mol Vis. 2010;16: 1640–1645. 20806036 PMC2927376

[11] Bailey JN, Loomis SJ, Kang JH, Allingham RR, Gharahkhani P, Khor CC, et al Genome-wide association analysis identifies TXNRD2, ATXN2 and FOXC1 as susceptibility loci for primary open-angle glaucoma. Nat Genet. 2016;48: 189–194. 10.1038/ng.3482 26752265 PMC4731307

[12] Rong SS, Lu SY, Matsushita K, Huang C, Leung CKS, Kawashima R, et al Association of the SIX6 locus with primary open angle glaucoma in southern Chinese and Japanese. Exp Eye Res. 2019;180: 129–136. 10.1016/j.exer.2018.12.014 30586556

[13] Li K, Yang C, Wan X, Xu J, Luo Q, Cheng Y, et al Evaluation of the association between five genetic variants and primary open-angle glaucoma in a Han Chinese population. Ophthalmic Genet. 2020;13: 1–5. 10.1080/13816810.2020.1747089 32281515

[14] Shiga Y, Akiyama M, Nishiguchi KM, Sato K, Shimozawa N, Takahashi A, et al Genome-wide association study identifies seven novel susceptibility loci for primary open-angle glaucoma. Hum Mol Genet. 2018;27: 1486–1496. 10.1093/hmg/ddy053 29452408 PMC6251544

[15] Wiggs JL and Pasquale LR. Genetics of glaucoma. Hum Mol Genet. 2017;26: R21–R27. 10.1093/hmg/ddx184 28505344 PMC6074793

[16] Bushati N and Cohen SM. microRNA functions. Annu Rev Cell Dev Biol. 2007;23: 175–205. 10.1146/annurev.cellbio.23.090506.123406 17506695

[17] Wang Y, Li F, Wang S. MicroRNA93 is overexpressed and induces apoptosis in glaucoma trabecular meshwork cells. Mol Med Rep. 2016;14: 5746–5750. 10.3892/mmr.2016.5938 27878244

[18] Zhang QL, Wang W, Alatantuya, Dongmei, Lu ZJ, Li LL, et al Down-regulated miR-187 promotes oxidative stress-induced retinal cell apoptosis through P2X7 receptor. Int J Biol Macromol. 2018;120: 801–810. 10.1016/j.ijbiomac.2018.08.166 30170060

[19] Zhang QL, Wang W, Li J, Tian SY, Zhang TZ. Decreased miR-187 induces retinal ganglion cell apoptosis through upregulating SMAD7 in glaucoma. Biomed Pharmacother. 2015;75: 19–25. 10.1016/j.biopha.2015.08.028 26463627

[20] Li G, Luna C, Qiu J, Epstein DL, Gonzalez P. Targeting of integrin beta1 and kinesin 2alpha by microRNA 183. J Biol Chem. 2010;285: 5461–5471. 10.1074/jbc.M109.037127 19940135 PMC2820774

[21] Luna C, Li G, Qiu J, Epstein DL, Gonzalez P. Role of miR-29b on the regulation of the extracellular matrix in human trabecular meshwork cells under chronic oxidative stress. Mol Vis. 2009;15: 2488–2497. 19956414 PMC2786891

[22] Ghanbari M, Darweesh SK, de Looper HW, van Luijn MM, Hofman A, Ikram MA, et al Genetic Variants in MicroRNAs and Their Binding Sites Are Associated with the Risk of Parkinson Disease. Hum Mutat. 2016;37: 292–300. 10.1002/humu.22943 26670097

[23] Ghanbari M, Sedaghat S, de Looper HW, Hofman A, Erkeland SJ, Franco OH, et al The association of common polymorphisms in miR-196a2 with waist to hip ratio and miR-1908 with serum lipid and glucose. Obesity (Silver Spring). 2015;23: 495–503. 25557604 10.1002/oby.20975

[24] Meola N, Gennarino VA, Banfi S. microRNAs and genetic diseases. Pathogenetics. 2009;2: 7 10.1186/1755-8417-2-7 19889204 PMC2778645

[25] Saus E, Soria V, Escaramis G, Vivarelli F, Crespo JM, Kagerbauer B, et al Genetic variants and abnormal processing of pre-miR-182, a circadian clock modulator, in major depression patients with late insomnia. Hum Mol Genet. 2010;19: 4017–4025. 10.1093/hmg/ddq316 20656788

[26] Hughes AE, Bradley DT, Campbell M, Lechner J, Dash DP, Simpson DA, et al Mutation altering the miR-184 seed region causes familial keratoconus with cataract. Am J Hum Genet. 2011;89: 628–633. 10.1016/j.ajhg.2011.09.014 21996275 PMC3213395

[27] Moschos MM, Droutsas K, Sioziou A, Dettoraki M, Gazouli M. Mutational Analysis of Pre-miR-184 and hsa-mir-568 in Greek Patients With Sporadic Keratoconus. Cornea. 2016;35: 631–633. 10.1097/ICO.0000000000000769 26845316

[28] Yu H, Liu Y, Bai L, Kijlstra A, Yang P. Predisposition to Behcet's disease and VKH syndrome by genetic variants of miR-182. J Mol Med (Berl). 2014;92: 961–967. 10.1007/s00109-014-1159-9 24801147

[29] Zhou Q, Hou S, Liang L, Li X, Tan X, Wei L, et al MicroRNA-146a and Ets-1 gene polymorphisms in ocular Behcet's disease and Vogt-Koyanagi-Harada syndrome. Ann Rheum Dis. 2014;73: 170–176. 10.1136/annrheumdis-2012-201627 23268366

[30] Ghanbari M, Iglesias AI, Springelkamp H, van Duijn CM, Ikram MA, Dehghan A, et al A Genome-Wide Scan for MicroRNA-Related Genetic Variants Associated With Primary Open-Angle Glaucoma. Invest Ophthalmol Vis Sci. 2017;58: 5368–5377. 10.1167/iovs.17-22410 29049738 PMC6110129

[31] Liu Y, Bailey JC, Helwa I, Dismuke WM, Cai J, Drewry M, et al A Common Variant in MIR182 Is Associated With Primary Open-Angle Glaucoma in the NEIGHBORHOOD Consortium. Invest Ophthalmol Vis Sci. 2016;57: 4528–4535. 10.1167/iovs.16-19688 27537254 PMC4991020

[32] Chatzikyriakidou A, Founti P, Melidou A, Minti F, Bouras E, Anastasopoulos E, et al MicroRNA-related polymorphisms in pseudoexfoliation syndrome, pseudoexfoliative glaucoma, and primary open-angle glaucoma. Ophthalmic Genet. 2018;39: 603–609. 10.1080/13816810.2018.1509352 30148417

[33] Greene LA, Liu DX, Troy CM, Biswas SC. Cell cycle molecules define a pathway required for neuron death in development and disease. Biochim Biophys Acta. 2007;1772: 392–401. 10.1016/j.bbadis.2006.12.003 17229557 PMC1885990

[34] Iglesias AI, Springelkamp H, van der Linde H, Severijnen LA, Amin N, Oostra B, et al Exome sequencing and functional analyses suggest that SIX6 is a gene involved in an altered proliferation-differentiation balance early in life and optic nerve degeneration at old age. Hum Mol Genet. 2014;23: 1320–1332. 10.1093/hmg/ddt522 24150847

[35] Abu-Amero KK, Kondkar AA, Mousa A, Almobarak FA, Alawad A, Altuwaijri S, et al Analysis of Cyclin-Dependent Kinase Inhibitor-2B rs1063192 Polymorphism in Saudi Patients with Primary Open-Angle Glaucoma. Genet Test Mol Biomarkers. 2016;20: 637–641. 10.1089/gtmb.2016.0140 27541204

[36] Ng SK, Casson RJ, Burdon KP, Craig JE. Chromosome 9p21 primary open-angle glaucoma susceptibility locus: a review. Clin Exp Ophthalmol. 2014;42: 25–32. 10.1111/ceo.12234 24112133

[37] Hu Z and He C. CDKN2B gene rs1063192 polymorphism decreases the risk of glaucoma. Oncotarget. 2017;8: 21167–21176. 10.18632/oncotarget.15504 28416752 PMC5400574

[38] Gutierrez-Camino A, Martin-Guerrero I, Garcia de Andoin N, Sastre A, Carbone Baneres A, Astigarraga I, et al Confirmation of involvement of new variants at CDKN2A/B in pediatric acute lymphoblastic leukemia susceptibility in the Spanish population. PLoS One. 2017;12: e0177421 10.1371/journal.pone.0177421 28481918 PMC5421813

[39] Liu Y and Allingham RR. Molecular genetics in glaucoma. Exp Eye Res. 2011;93: 331–339. 10.1016/j.exer.2011.08.007 21871452 PMC4293633

[40] Li G, Luna C, Qiu J, Epstein DL, Gonzalez P. Alterations in microRNA expression in stress-induced cellular senescence. Mech Ageing Dev. 2009;130: 731–741. 10.1016/j.mad.2009.09.002 19782699 PMC2795064

[41] Li WQ, Pfeiffer RM, Hyland PL, Shi J, Gu F, Wang Z, et al Genetic polymorphisms in the 9p21 region associated with risk of multiple cancers. Carcinogenesis. 2014;35: 2698–705. 10.1093/carcin/bgu203 25239644 PMC4247519

[42] Wang X, Li W, Ma L, Gao J, Liu J, Ping F, et al Association study of the miRNA-binding site polymorphisms of CDKN2A/B genes with gestational diabetes mellitus susceptibility. Acta Diabetol. 2015;52: 951–958. 10.1007/s00592-015-0768-2 25990668

[43] Horswell SD, Fryer LG, Hutchison CE, Zindrou D, Speedy HE, Town MM, et al CDKN2B expression in adipose tissue of familial combined hyperlipidemia patients. J Lipid Res. 2013;54: 3491–3505. 10.1194/jlr.M041814 24103848 PMC3826695

[44] Cao D, Jiao X, Liu X, Hennis A, Leske MC, Nemesure B, et al CDKN2B polymorphism is associated with primary open-angle glaucoma (POAG) in the Afro-Caribbean population of Barbados, West Indies. PLoS One. 2012;7: e39278 10.1371/journal.pone.0039278 22761751 PMC3384655

[45] Dimasi DP, Burdon KP, Hewitt AW, Fitzgerald J, Wang JJ, Healey PR, et al Genetic investigation into the endophenotypic status of central corneal thickness and optic disc parameters in relation to open-angle glaucoma. Am J Ophthalmol. 2012;154: 833–842 e2. 10.1016/j.ajo.2012.04.023 22840486

[46] Mabuchi F, Sakurada Y, Kashiwagi K, Yamagata Z, Iijima H, Tsukahara S. Association between genetic variants associated with vertical cup-to-disc ratio and phenotypic features of primary open-angle glaucoma. Ophthalmology. 2012;119: 1819–1825. 10.1016/j.ophtha.2012.02.044 22584021

[47] Micheal S, Ayub H, Khan MI, Bakker B, Schoenmaker-Koller FE, Ali M, et al Association of known common genetic variants with primary open angle, primary angle closure, and pseudoexfoliation glaucoma in Pakistani cohorts. Mol Vis. 2014;20: 1471–1479. 25489222 PMC4225136

[48] Osman W, Low SK, Takahashi A, Kubo M, Nakamura Y. A genome-wide association study in the Japanese population confirms 9p21 and 14q23 as susceptibility loci for primary open angle glaucoma. Hum Mol Genet. 2012;21: 2836–2842. 10.1093/hmg/dds103 22419738

[49] Philomenadin FS, Asokan R, N V, George R, Lingam V, Sarangapani S. Genetic association of SNPs near ATOH7, CARD10, CDKN2B, CDC7 and SIX1/SIX6 with the endophenotypes of primary open angle glaucoma in Indian population. PLoS One. 2015;10: e0119703 10.1371/journal.pone.0119703 25798827 PMC4370747

[50] Ghanbari M, Franco OH, de Looper HW, Hofman A, Erkeland SJ, Dehghan A. Genetic Variations in MicroRNA-Binding Sites Affect MicroRNA-Mediated Regulation of Several Genes Associated With Cardio-metabolic Phenotypes. Circ Cardiovasc Genet. 2015;8: 473–486. 10.1161/CIRCGENETICS.114.000968 25814643

